# A Web-Based eHealth Intervention to Improve the Quality of Life of Older Adults With Multiple Chronic Conditions: Protocol for a Randomized Controlled Trial

**DOI:** 10.2196/25175

**Published:** 2021-02-19

**Authors:** David H Gustafson Sr, Marie-Louise Mares, Darcie C Johnston, Jane E Mahoney, Randall T Brown, Gina Landucci, Klaren Pe-Romashko, Olivia J Cody, David H Gustafson Jr, Dhavan V Shah

**Affiliations:** 1 Center for Health Enhancement Systems Studies University of Wisconsin–Madison Madison, WI United States; 2 Department of Industrial and Systems Engineering University of Wisconsin–Madison Madison, WI United States; 3 Department of Communication Arts University of Wisconsin–Madison Madison, WI United States; 4 Department of Medicine University of Wisconsin–Madison Madison, WI United States; 5 Department of Family Medicine University of Wisconsin School of Medicine & Public Health Madison, WI United States; 6 School of Journalism and Mass Communication University of Wisconsin–Madison Madison, WI United States

**Keywords:** eHealth, telemedicine, aged, geriatrics, multiple chronic conditions, depression, social support, quality of life, primary care, health expenditures, mobile phone

## Abstract

**Background:**

Multiple chronic conditions (MCCs) are common among older adults and expensive to manage. Two-thirds of Medicare beneficiaries have multiple conditions (eg, diabetes and osteoarthritis) and account for more than 90% of Medicare spending. Patients with MCCs also experience lower quality of life and worse medical and psychiatric outcomes than patients without MCCs. In primary care settings, where MCCs are generally treated, care often focuses on laboratory results and medication management, and not quality of life, due in part to time constraints. eHealth systems, which have been shown to improve multiple outcomes, may be able to fill the gap, supplementing primary care and improving these patients’ lives.

**Objective:**

This study aims to assess the effects of ElderTree (ET), an eHealth intervention for older adults with MCCs, on quality of life and related measures.

**Methods:**

In this unblinded study, 346 adults aged 65 years and older with at least 3 of 5 targeted high-risk chronic conditions (hypertension, hyperlipidemia, diabetes, osteoarthritis, and BMI ≥30 kg/m2) were recruited from primary care clinics and randomized in a ratio of 1:1 to one of 2 conditions: usual care (UC) plus laptop computer, internet service, and ET or a control consisting of UC plus laptop and internet but no ET. Patients with ET have access for 12 months and will be followed up for an additional 6 months, for a total of 18 months. The primary outcomes of this study are the differences between the 2 groups with regard to measures of quality of life, psychological well-being, and loneliness. The secondary outcomes are between-group differences in laboratory scores, falls, symptom distress, medication adherence, and crisis and long-term health care use. We will also examine the mediators and moderators of the effects of ET. At baseline and months 6, 12, and 18, patients complete written surveys comprising validated scales selected for good psychometric properties with similar populations; laboratory data are collected from eHealth records; health care use and chronic conditions are collected from health records and patient surveys; and ET use data are collected continuously in system logs. We will use general linear models and linear mixed models to evaluate primary and secondary outcomes over time, with treatment condition as a between-subjects factor. Separate analyses will be conducted for outcomes that are noncontinuous or not correlated with other outcomes.

**Results:**

Recruitment was conducted from January 2018 to December 2019, and 346 participants were recruited. The intervention period will end in June 2021.

**Conclusions:**

With self-management and motivational strategies, health tracking, educational tools, and peer community and support, ET may help improve outcomes for patients coping with ongoing, complex MCCs. In addition, it may relieve some stress on the primary care system, with potential cost implications.

**Trial Registration:**

ClinicalTrials.gov NCT03387735; https://www.clinicaltrials.gov/ct2/show/NCT03387735.

**International Registered Report Identifier (IRRID):**

DERR1-10.2196/25175

## Introduction

### Background

Multiple chronic conditions (MCCs) are both common among patients aged 65 years and older and expensive to manage. Two-thirds of Medicare beneficiaries have more than one chronic condition, such as diabetes or high blood pressure, and they account for more than 90% of all Medicare spending [1-3]. According to the latest available numbers from the Centers for Medicare and Medicaid Services, per-capita spending in 2017 increased exponentially with the number of chronic conditions, from US $2032 for patients without MCCs to US $32,247 for patients with 6 conditions or more. Patients with ≥6 chronic conditions, making up just 17% of beneficiaries, accounted for 53% of expenditures [1]. In summary, the impact of MCCs on health care use and costs is immense.

For the individual patient, MCCs are equally consequential. MCCs are associated with lower quality of life, poorer response to treatment, worse medical and psychiatric outcomes, higher mortality, and greater financial burden for both patients and families [4]. In addition, numerous studies indicate that chronic conditions contribute to loneliness and that loneliness in turn contributes to reduced functionality and chronic illness [5-7].

MCCs are not simply aggregates of several distinct conditions. They represent overlapping conditions that often have common root causes and, when grouped together, can severely impact a patient’s treatment options as well as quality of life. Primary care providers face many challenges in treating patients with MCCs, particularly how to address the complexity and chronic nature of MCCs within the constraining time frames typically allotted in primary care settings [8-12]. As such, most providers necessarily focus on managing medication and laboratory results for MCCs, with little time left for self-management strategies and skills [13]. However, treatment adherence, health tracking, and feedback to clinicians are likely to be particularly important for patients with MCCs, given the challenges of polypharmacy and multiple ongoing treatment needs. In addition, patients need education about how to *live* with their conditions, in that they are chronic.

Previous studies have shown that information and communication technologies can address these gaps, improving not only self-management and health care effectiveness but also social and emotional support. An extensive review of eHealth apps for cardiovascular disease [14] found promising results in clinical trials on hypertension and hyperlipidemia; another review [15] found that eHealth interventions reduce blood pressure and increase the likelihood of blood pressure control. Other reviews [16,17] found positive outcomes in 29 of 32 studies of chronic condition interventions delivered via computer and mobile phone, with more impact coming from multiservice programs [18]. Internet-based interventions have proven effective in reducing pain [19,20]. Finally, a review of eHealth programs for diabetes concluded that there is clear potential for benefit, although studies have generally been poorly designed or underpowered [21].

### Need for a Trial

This paper reports on the study design and methods of a trial of ElderTree (ET), a web-based health intervention designed to improve quality of life and socioemotional outcomes among older adults with MCCs. One of several eHealth systems collectively known as CHESS (Comprehensive Health Enhancement Support System), ET is an information and support platform developed by our Agency for Healthcare Research and Quality Center of Excellence in Active Aging to help older adults remain independent. ET was previously tested in a randomized controlled trial (RCT) involving 390 older adults in 5 Wisconsin counties (urban, suburban, and rural) who were followed up during the 12 months of the intervention [22].

As reported in a paper submitted for publication and under review, the results of the intention-to-treat RCT showed that study arm interacted with amount of primary care use to predict mental quality of life, social support (both received and provided), and depression, such that for participants with 3 or more primary care visits in the 6 months before baseline, those in the ET group performed significantly better than those in the control group. In addition, positive results among ET participants for functional independence, as measured by independent activities of daily living, approached significance.

The results of the RCT suggest that ET may be more effective among patients dealing with MCCs, given that primary care use is relatively high among such patients, and that a system such as ET may be most effective if integrated into primary care. The study described in this protocol seeks to build on those findings, examining effects among patients in primary care with MCCs rather than a general older population, focusing not only on patient-centered outcomes such as quality of life but also on laboratory scores and levels of crisis-related health care use.

### Choice of Comparators

**Figure 1:**
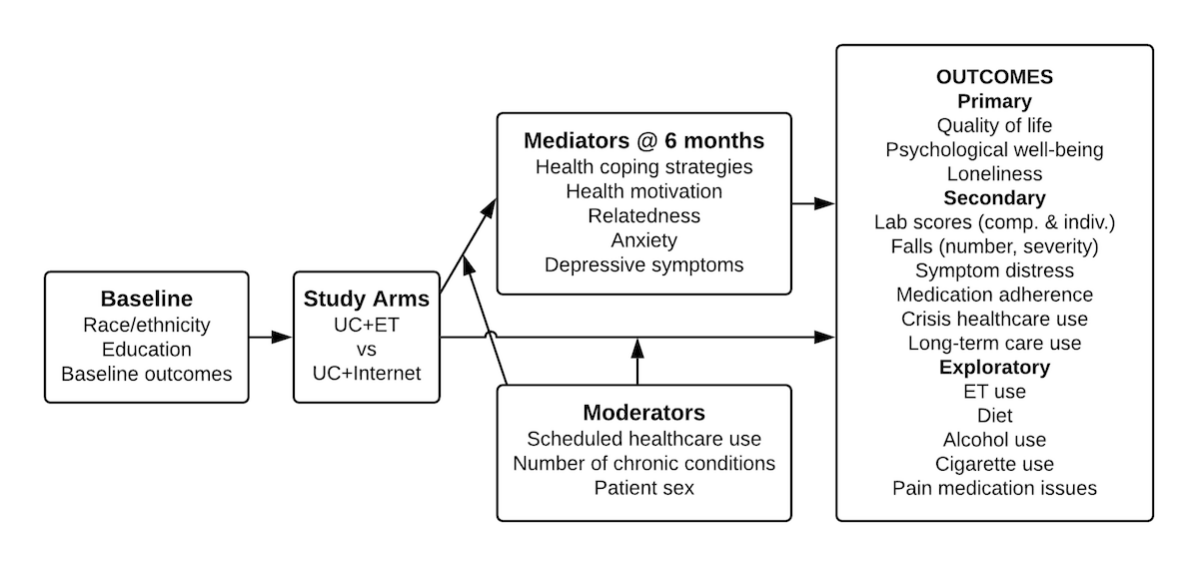
Study logic diagram. ET: ElderTree; UC: usual care.

Participants recruited from primary care clinics have been randomized to receive either (1) usual care (UC) plus a touchscreen laptop delivering the ET intervention (UC+ET) or (2) a touchscreen laptop delivering internet access and links to high-quality medical information websites but not to ET (UC+internet) for 12 months. This comparison controls for the effects of access to the laptop and the internet, isolating the specific effects of access to ET. The overarching goal of the study is to test the effects of ET on patient outcomes, including the examination of mediating processes and subgroup differences (moderation). Figure 1 shows the logic diagram.

### Study Objectives

#### Primary Objective

The primary objective is to determine whether patients assigned to UC+ET (vs those assigned to UC+internet) will have greater improvements over time in quality of life and psychological well-being and greater reductions over time in loneliness. Quality of life is a multifaceted variable encompassing global assessments of health and quality of life as well as physical, mental, emotional, and social dimensions. Psychological well-being encompasses feelings of meaningfulness, social connectedness, engagement, and optimism.

#### Secondary Objectives

There are several key secondary objectives. One is to determine whether patients assigned to UC+ET, versus UC+internet, will have greater improvements in composite and individual health scores (see the *Measures* section). Other secondary objectives are to determine whether patients assigned to UC+ET will have greater reductions in the number and severity of falls, greater improvements in symptom distress, greater improvements in medication adherence, and greater reductions in crisis health care use and long-term care use.

#### Exploratory

We plan to explore the effects of study arm on improvements over time in patients’ diet, alcohol use, cigarette use, and pain medication issues (eg, hoarding). Within the UC+ET arm, we will describe the amount and types of ET use (ie, services used) and examine the associations between these variables and the primary outcomes.

#### Mediation

We will investigate whether the effects of study arm on change from baseline to endpoint in primary and secondary outcomes (Figure 1) are mediated by midpoint (6-month) changes in health coping strategies, health-related motivation, feelings of relatedness, and levels of anxiety and depressive symptoms.

#### Moderation

We will investigate whether the effects of study arm on change from baseline to endpoint in primary and secondary outcomes (Figure 1) are moderated by participant sex (ie, women show more benefits than men), scheduled health care use (those with higher levels of primary, specialist, physical and occupational therapy, chiropractor, and counseling visits show more benefits), and number of chronic conditions (those with more conditions show more benefits).

### Trial Design

The ET trial is a randomized controlled design with 2 parallel groups with a 1:1 allocation.

## Methods

### Sample Size and Study Setting

A total of 346 older adult patients with at least 3 of 5 targeted high-risk chronic conditions (hypertension, hyperlipidemia, diabetes, osteoarthritis, and BMI ≥30 kg/m^2^) have been recruited from primary care clinics within the University of Wisconsin–Madison Department of Family Medicine and General Internal Medicine system (UW Health).

### Eligibility Criteria

Eligible patients (1) are aged 65 years or older; (2) have been treated in the clinic for at least the previous 18 months (to have baseline laboratory data on all measures) with no plans to leave during the study period; (3) have 3 or more of the following 5 chronic conditions: hypertension, hyperlipidemia, diabetes, arthritis, and BMI ≥30 kg/m^2^; (4) report no current psychotic disorder that would prevent participation; (5) have no acute medical problem requiring immediate hospitalization; (6) do not have a visual or motor impairment that prevents them from using a computer; (7) are able to read and sign the consent form in English; (8) are willing to share health-related study data (eg, laboratory scores, health care utilization); (9) allow researchers to share information with the patient’s primary care physician; and (10) do not have moderate or advanced dementia. In addition to 3 or more of the 5 conditions mentioned earlier, patients may have any of the following conditions: chronic kidney disease, chronic pain, chronic obstructive pulmonary disease, congestive heart failure, arrhythmia or atrial fibrillation, pulmonary heart or vascular disease, anxiety, and depression. We will document and describe eligible people who choose not to participate.

### Intervention Groups

Patients in both conditions are continuing with their UC provided by primary care and internal medicine clinics in the UW system. Participants receive the intervention for 12 months and are followed up 6 months later for a total on-study period of 18 months.

All participants are offered a study laptop, whether or not they have one. A computer they already own may be older and out of date, so using the study laptop is better for both participant and technical support. In addition, the laptop can be a dedicated computer for the study so that participants are not sharing the study device with others in the household.

#### Control Condition: UC+Internet

In addition to UC, patients in the control condition receive internet service and a laptop computer, provided by the study group if desired, as well as training for 12 months. General health information websites published by the Cleveland Clinic, National Institute on Aging, American Academy of Family Physicians, and Mayo Clinic are loaded on the computer for easy access. These sites are vetted for quality by our research team. We expect the UC+internet intervention to be relatively ineffective because information alone is unlikely to have a significant effect on health behaviors [23-26]. Instead, access to the device, the internet, and the sites will function both as an attention control and as a way to isolate the specific effects of access to ET. In summary, this study is designed with an attention control rather than a pure control comparison.

#### Experimental Condition: UC+ET

Patients in the experimental condition receive ET access for 12 months in addition to their UC, as well as a laptop computer and internet, if desired. These patients do not receive the 4 health information websites placed on computers for the control condition patients, although they could seek them out.

### ET Intervention

For more than 30 years, our Center has been developing and testing a suite of evolving eHealth systems built on principles of continuing care and self-management: long duration [27]; assertive outreach [28]; tracking [29]; prompts [30]; action planning [31]; problem solving [13]; self-tailoring [13]; peer, family, and clinical support [32]; case management [33]; and care coordination [34]. In randomized trials, these CHESS systems significantly improved asthma control [35]; quality of life and cost of care in patients with HIV [36]; quality of life and self-efficacy in patients with breast cancer, including older adult women [37], compared with control [38] and internet [26] groups; risky drinking [39]; and caregiver burden, symptom distress, and median length of survival in patients with lung cancer [40].

Although ET is built on the CHESS experience, its interface and content are quite different from our systems serving other health concerns and populations. ET is designed specifically for older adults and with their input, featuring larger fonts, fewer options, appealing images and layouts, and uncluttered screens for easier comprehension, navigation, and usability [41].

#### System Overview

ET provides tools, motivation, and social support to help patients (1) manage their specific set of chronic conditions, (2) communicate with peers and research staff, and (3) improve communication with clinicians. This study is based on the earlier ET system, with a few new or enhanced services (weekly health tracking survey, clinician report, daily entertainment feature) and expanded health information resources. The design and navigation are based on the original ET system and the principles established in our earlier testing [41].

As stated earlier (see the *Need for a Trial* section), our original clinical trial found the greatest improvements associated with ET in psychosocial outcomes. To further these outcomes, we added a daily interactive entertainment feature (lighthearted polls, quizzes, games, and reflection prompts) as a means of boosting enjoyment of the site and engagement with other participants.

The weekly survey is an enhancement of a basic health tracking feature in the original version, and the related clinician report, both described below, is altogether new to ET. We used a clinician report in a lung cancer RCT comparing a CHESS system alone with CHESS+clinician report [42] and found that the addition improved (*P*<.001) symptom distress by over 100% (26.2% improvement with CHESS vs 53% in those with CHESS+clinician report: n=71 vs n=68, respectively).

#### Weekly Survey

Patients using ET are prompted to complete weekly check-ins, on which they rate their experience on 10 health indicators: sleep, nutrition, physical activity, cognition, balance, falls, mood, pain management, medication adherence, and quality of social interactions. At the completion of each check-in, ET commends positive results or, if struggle is detected, directs the patient to helpful site information. The system may also recommend contacting the clinic if the algorithm detects a sudden or steep change or a problem that may not be severe but is not improving. In addition, for easy visual interpretation, ET displays a graph charting the patient’s responses for each indicator over the last 3 months. The graph, showing health trends and current status, is also shared with the primary care clinic (the clinician report). In addition, ET offers the patient a printout to take to a primary care visit, in case the clinician has not viewed the report.

#### Clinician Report

MCCs can lead to rapid declines in health [2]. However, support for patients with MCCs usually consists of periodic, onsite contact with primary care clinicians, who may be unaware of and/or cannot respond as promptly as may be warranted to such changes. Moreover, patients may avoid “bothering” the doctor, foreclosing a source of help that might make a difference.

The clinician report shares timely information on patient general indicators and helps both patients and clinicians prepare for and make the most of primary care office visits. As a one-page graphic summary of the patient’s health tracking data, the report can be viewed in a matter of seconds, avoiding a time burden for clinicians while allowing them to be better informed and provide treatment more responsively on the basis of patient needs.

One week before a participant’s scheduled appointment, the clinician report is sent to the primary care doctor via email (password-protected PDF file) or fax. The mode of delivery has been chosen by each clinic based on what works best in their workflow. Typically, the recipient of the clinician report is the clinic manager or other administrator, who then forwards it to the clinician, either printing it out or via email. A hard copy of the clinician report is also mailed directly to the participant, as noted earlier, to take to the appointment.

In addition, every 2 months, the project manager prepares a clinician report summary that provides an overview for all patients at each clinic. The goal is to help clinicians identify patients who are reporting issues between appointments, such as missed medications or mood declines, particularly as patients continue to isolate due to COVID-19. This report is emailed to clinic managers to share with individual clinicians.

#### Theoretical Foundation

Similar to other CHESS systems, ET is consistent with Self-Determination Theory, which asserts that satisfying 3 fundamental psychological needs contributes to adaptive functioning: competence (feeling effective), social relatedness (feeling connected to others), and autonomy (feeling internally motivated rather than coerced) [43].

#### Interface and Features

The key features of the site, aligned with how they relate to Self-Determination Theory, are described in Table 1. The ET system is based on the 3 components of the theory as shown, but we acknowledge that these are interrelated, potentially larger, latent constructs [44] and that services for one outcome likely affect other outcomes.

**Figure 2:**
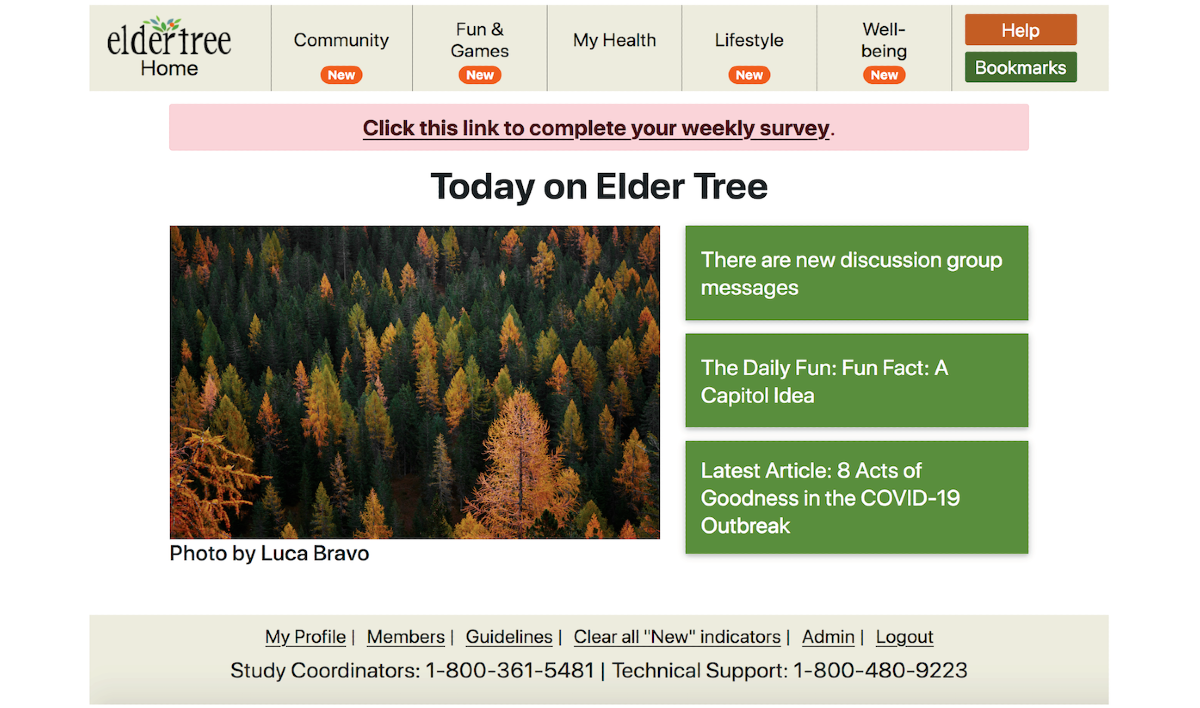
ElderTree homepage view.

Figure 2 shows the ET home page, with its clear navigation to features listed in Table 1 and prompts customized to the individual user.

**Table 1:** Key features of the ElderTree eHealth intervention.

Aim of feature and ElderTree tab or element		Feature title or function	Description
**Tools fostering health-related coping competence**			
	My Health	Health Library	Informational resources and materials, organized by health and wellness topic area
	My Health	Weekly survey, clinician report	Tracking of 10 self-reported general health measures, graphed over time, displayed for patients, and sent to primary care clinicians
	Community	Health Matters discussion board	Tips, experiences, and resources for managing multiple chronic conditions [45]
**Tools fostering social relatedness and positive affect**			
	Community	Discussion Groups	Monitored online support and chat forums [46,47]
	Community	Private Messages	Email-like function for private communication among users and research staff
	Community	Bulletin Board	Local activities, continuously updated
	Fun & Games	Laugh Out Loud, Social Games, Daily Fun	Interactive games, jokes, puzzles, polls, quizzes, videos, and trivia, refreshed daily
	All areas	Comment functionality	Universal posting function to encourage engagement, support, and relationship building
	Footer of every page	Members profiles	User-created interest and history profiles serving as introductions
**Tools fostering health-related motivation**			
	Well-being	Daily Reflection	Journal function with prompts based on positive psychology principles
	Opening screen, Community	Thought of the Day	Motivational and inspirational quotes
	Well-being	Relaxation Exercises	Progressive relaxation, deep breathing, meditation, and mindfulness audio and video
	Lifestyle	Lifestyle blog	Inspirational articles on topics such as travel, mind and body, and nature

### Measures

Table 2 lists the variables and measures for primary, secondary, and exploratory outcomes. Scales were selected to have good psychometric properties with similar populations. Patient-reported outcome measures, including any modifications to validated scales, are described following the table.

**Table 2:** Study outcomes, variables, and measures.

Category and variable			Measure name and description		Number of items		Source
**Primary outcomes**							
	Quality of life	PROMIS^a^ Global Health		10		Patient	
	Psychological well-being	Psychological Flourishing Scale		8		Patient	
	Loneliness	UCLA Loneliness Scale		8		Patient	
**Secondary outcomes**							
	Composite and individual laboratory scores	Z scores: mm Hg, mg/dL, glycated hemoglobin (HbA_1c_), Visual Analog Scale pain, BMI deviation from normal range		6		EHR^b^	
	Falls	Number/severity in past 3 months		2		Patient	
	Symptom distress	General Symptom Distress Scale, Bayliss Disease Burden Scale		21		Patient	
	Medication adherence	Based on Brief Medication Questionnaire		8		Patient	
	Crisis health care use	Emergency room visits, urgent care visits, days/occurrences in hospital, 30-day readmissions		5		EHR, patient	
	Long-term care use	Nights in assisted living, nursing home		2		Patient	
**Exploratory outcomes**							
	Amount or type of ElderTree use	Automatically logged keystrokes		N/A^c^		System logs	
	Diet	Healthy foods and snacks (custom list)		7		Patient	
	Alcohol use	Alcohol Use Disorders Identification Test		8		Patient	
	Cigarette use	Cigarettes per day		1		Patient	
	Pain medication issues	Centers for Disease Control and Prevention pain medication survey (modified)		8		Patient	

^a^PROMIS: Patient-Reported Outcomes Measurement Information System.

^b^EHR: electronic health record.

^c^N/A: not applicable.

#### Primary Outcomes

Quality of life is assessed using the Patient-Reported Outcomes Measurement Information System (PROMIS) Global Health measure [48,49]. For consistency with other measures (ie, to reduce respondent burden), the time frame has been modified to refer to the past 2 weeks. Psychological well-being is assessed using the Psychological Flourishing Scale [50]. The wording of items has been somewhat modified, including simplifying double-barreled items. For example, “I lead a purposeful and meaningful life” is shortened to “I lead a meaningful life.” Loneliness is measured using the 8 items from the UCLA Loneliness Scale with the highest factor loadings among older adults in the validation paper by Russell [51].

#### Secondary Outcomes

Health scores are obtained from the patient’s electronic health record (EHR). Composite health scores are the averaged Z scores of mm Hg for hypertension, mg/dL for hyperlipidemia, glycated hemoglobin (HbA_1c_) for diabetes, deviation of BMI from the normal range of 18.5-24.9 kg/m^2^, and Visual Analog Scale [52] pain ratings. Each of these variables will also be examined separately. Falls are assessed with 2 items asking how often the participant had fallen in the past 3 months and how many of the falls required medical attention. A fall is defined in the survey as “the body going to the ground without being pushed.” Symptom distress is assessed using a combined list of symptoms and chronic conditions from the General Symptom Distress Scale [53] and Bayliss Disease Burden Scale [54], assessing the severity of distress for each over the past 2 weeks (0=do not have this, 1=not very distressing, and 5=extremely distressing). Medication adherence is assessed with 8 items, 6 based on the Brief Medication Questionnaire by Svarstad et al [55]. We simplified response options so that participants rate how often they had specific issues with medication (1=never and 5=always). On the basis of patients’ experiences, we added 2 original items, “Feels like I no longer need it” and “Feels like I don’t need the full dose.” Patients also report on crisis health care (number of urgent care visits, emergency room visits, and hospitalizations, plus the number of days of each hospitalization, with the latter 2 items used to calculate the number of 30-day hospital readmissions). In addition, patients report on the use of long-term care (number of nights spent in assisted living facilities and nursing homes) over the past 3 months.

#### Exploratory Outcomes

Participants rate 7 items about their diet, indicating how often (1=never and 5=every day) they consumed healthy (eg, vegetables) and unhealthy (eg, processed or sugary) foods. They reported on problem drinking using items 3 to 10 of the Alcohol Use Disorders Identification Test [56] and number of cigarettes smoked per day. They report on pain medication issues using a modified, 8-item version of the Centers for Disease Control Pain Medication Survey [57]. ET usage data are collected automatically, including when a patient accesses ET, services used, and duration of use.

#### Mediators

Anxiety is assessed using the Generalized Anxiety Disorder (GAD-7) scale [58]. Depressive symptoms are assessed using the Patient Health Questionnaire Depression Scale (PHQ-8) [59]. The response options for all items in both scales are frequency (1=not at all and 4=nearly every day) in the past 2 weeks. Health coping strategies are assessed using 10 items from the Ways of Coping Scale [60]. Relatedness is assessed with the McTavish Bonding Scale [61] plus the 3 items from the short form of the PROMIS emotional support scale [62]. For all 9 items, patients indicate the frequency of particular types of support (eg, someone you can count on to listen to you when you need to talk; 1=never and 5=always). Health motivations are assessed with 2 items from the autonomous motivation subscale and 2 from the external regulation subscale of the Treatment Self-Regulation Questionnaire [63].

#### Moderators

Patients report on their scheduled health care use by indicating the number of visits to primary care, specialists, physical and occupational therapists, chiropractors, and counseling. For a number of chronic conditions, they check off applicable items from a list of 27 conditions [64] and write in additional diagnoses if necessary. Participants also indicate their sex.

#### Covariates

Patients rate their comfort using technology (0=do not know what this is, 1=very uncomfortable, and 5=very comfortable) for 6 communication technologies (eg, computer). For physical issues with technology, they use a checklist to indicate issues with 5 items each for a computer or tablet and a smartphone (eg, vision, hand pain, or tremors). To gauge emotional well-being, patients check a list of 15 possible life stressors from the Social Readjustment Rating Scale [65]. Participants also report their ethnicity and race, education, income level, health insurance type (checklist including Medicare, Medicaid, ObamaCare, military, private insurance, other person’s insurance, no insurance, and other), whether they have a significant other, their housing type (own home, rent, live in someone else’s home, assisted living, residential care, nursing home, and other), and whether they live alone or with someone else.

### Timeline

Recruitment was conducted from January 2018 to December 2019; the intervention period will end in June 2021. Table 3 shows the timeline by year of the study; Year 1 began on April 1, 2017, and Year 5 will end on March 31, 2022. Patients will be tracked for 12 months with access to the interventions plus 6 months for follow-up for a total of 18 months.

**Table 3:** Timeline of project activities.

Activity	Timeline
Clinicians set thresholds and comment on content	Year 1, months 1-3
Adapt ElderTree; prepare laptops and study materials	Year 1, months 1-9
Finalize outcome surveys	Year 1, months 4-9
Prepare clinics for ElderTree study	Year 1, months 4-9
Receive institutional review board approval	Year 1, months 7-9
Recruit, pretest, and randomize patients	Year 1, month 10, to year 3, month 9
Refresh ElderTree content	Year 1, month 10, to year 5, month 3
Collect quantitative and qualitative data	Year 1, month 10, to year 5, month 6
Clean and prepare data	Year 1, month 10, to year 5, month 6
Analyze results	Year 3, month 6, to year 5, month 12
Publish	Year 3, month 12, to year 5, month 12

### Power Analyses for Primary Outcomes

We focus on the effect of Cohen *d*=0.50 on the primary outcome of patients’ perceptions of their health-related quality of life, given recommendations that this is the minimally important difference for quality-of-life measures in clinical trials [66]. Our other primary outcomes are loneliness and psychological well-being. Here, effect sizes tend to be smaller. For example, a prior web-based intervention for rural women with chronic diseases showed an effect of Cohen *d*=0.29 on loneliness among those who scored above the median on baseline loneliness, depression, and stress [67]. Given that our intervention is substantially longer (12 months vs 22 weeks) and has more components specifically designed to address social connectedness, we expect somewhat larger effects; however, we do not expect to reach Cohen *d*=0.50. Balancing the need to be adequately powered with the need to focus on meaningful impacts, we have powered the study to detect a main effect of Cohen *d*=0.35 for our primary outcomes. Adequate power to detect a between-subjects effect of Cohen *d*=0.35 with a 4-time-point repeated measures multivariate analysis of variance (1−β=.80; α=.05) will require a final sample of 262 patients (130 per arm) [68-73]. On the basis of our prior trial of ET, we assumed 20.5% attrition and thus arrived at the recruitment goal of 330 patients.

### Recruitment

The UW Clinical Research Data Service (CRDS) identified from clinic records those patients meeting the inclusion criteria. Potential UW Health participants received an opt-in letter from the university’s Office of Clinical Trials. The letter described the study and included a postage-paid return invitation for further contact with the study team.

Study staff called potential participants who opted in to provide a detailed study overview, including benefits and potential risks of participation. If interested in the study, patients were asked additional questions regarding eligibility that were not addressed in the clinic record.

Patients who verbally confirmed they wanted to be in the study and met the screening criteria were mailed the baseline survey and received a home visit from a member of the research team, at which time written consent was obtained, completed baseline was collected, and randomization was determined.

### Randomization

**Figure 3:**
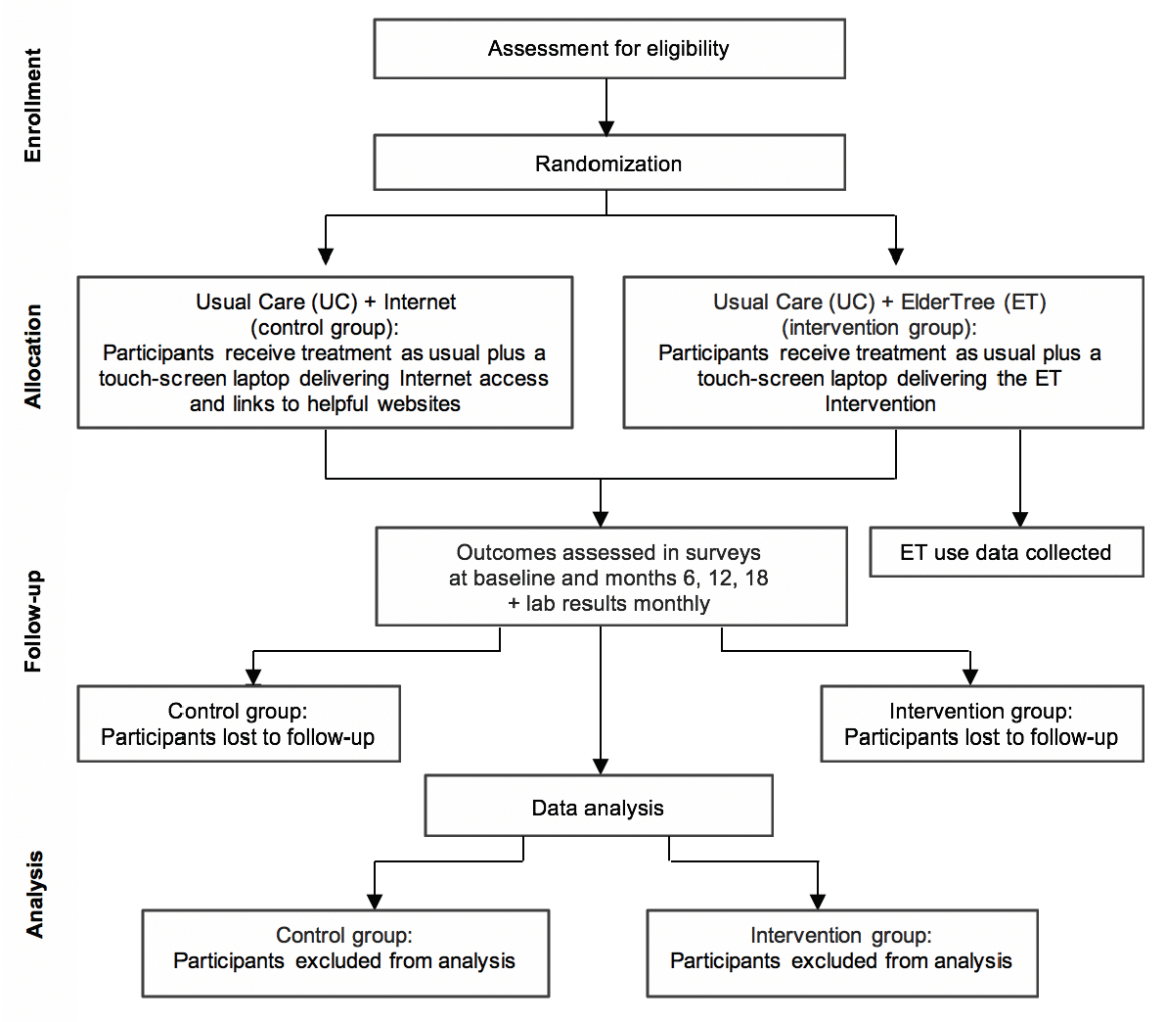
CONSORT (Consolidated Standards of Reporting Trials) flow diagram.

The project manager used a computer-generated allocation sequence to randomize patients on a 1:1 ratio to the experimental (UC+ET) or control (UC+internet) group, stratified by sex, clinic site, and number of chronic conditions (3-5 vs 6+). When baseline assessment and consent were complete, the research staff conducted equipment setup and training based on group assignment provided in a sealed opaque envelope. Once the assignment was made, participants could not be blinded to their condition, given that those in the ET arm are asked to participate in the site for the duration of the intervention and those in the control arm are not. The researcher doing the training also could not be blinded to the condition after assignment. Figure 3 shows the flow of participants through the trial.

### Data Sources and Collection

#### EHRs

Health care use, laboratory scores, and chronic conditions are gathered from EHRs. The UW CRDS pulls participant EHR data and shares the data with the study team via Research Electronic Data Capture (REDCap).

#### Patient Surveys

Patient-reported measures are gathered via participant surveys at months 0, 6, and 12 and an 18-month follow-up; demographics were gathered only at baseline. Each participant survey is expected to take 20 to 30 min. Surveys are mailed to participants with a stamped return envelope. Survey data are entered into REDCap. Participants are paid US $10 for each completed survey.

#### ET System Data

ET use data are automatically collected in time-stamped log files by subjects’ ET code number, including when ET is accessed, services used, duration of use, pages viewed, messages posted versus received, and content of messages.

#### Qualitative Interviews

In-depth phone interviews will be conducted with random samples of participants assigned to the ET condition (not the attention control) early in the intervention period (1-4 months), mid-study (5-6 months), and at end of study (10-12 months). In addition, participants from racial or ethnic minorities will be interviewed. Our goal is 10 to 12 participants in each of these 4 groups (early, mid, and end of study, and minorities). Patients will be asked about barriers to use and the challenges and benefits of ET. Early and midpoint interviews help identify issues (eg, confusion, technical challenges) to be addressed with the ET group as a whole. End-of-study interviews shed light on the barriers, benefits, and contexts of ET use to inform future work. Interviews are expected to last 30 to 60 min and are based on a standard set of questions, although clarification questions may vary. We will seek a mix of men versus women, number of chronic conditions, and clinic sites. Potential participants will be contacted by ET system messaging or by phone. All interviews will be transcribed for more detailed coding, including quantitative tagging of key concepts.

### Retention

Retention is promoted by providing ready access to support for patients’ use of the technologies and by actively following up with patients to encourage them to return surveys. If a survey is not returned within 2 weeks, a research team member calls to check that the survey was received and encourages the patient to complete and return it in the addressed stamped envelope. The date and time of the phone call are recorded in REDCap, along with information gathered during the conversation and whether the researcher talked to the participant directly or left a message. If we cannot reach the participant, another copy of the survey is sent with a personal note asking them to complete it or call our toll-free number if they have questions or are no longer interested. In the prior ET study, survey response rates were 90.5% at 6 months and 79.5% at 12 months. If patients drop out, we do not use their EHR data beyond their dropout point.

### Data Management

To mitigate the risk of breaches of patient confidentiality, all subjects are assigned a unique code number. All contact information and survey data are housed electronically in REDCap. Survey data are double-entered by 2 different individuals to ensure accuracy. Paper-based files are stored in a locked room in locked file cabinets and can be accessed only by authorized personnel. Participant EHR data are shared by the UW CRDS with the study team via REDCap. The database administrator provides access to study data at appropriate levels for various members of the research team. Members of the research team are able to view deidentified individual and clinic-level aggregations of variables.

### Statistical Methods

#### Statistical Assumptions

Parametric test assumptions such as normality, linearity, homoscedasticity or homogeneity of variance, and missing data patterns will be assessed as follows.

#### Predictor Assumptions

Successful randomization of participants will be tested based on sex, clinic site, number of chronic conditions, and demographics, including all planned covariates (see the logic diagram in Figure 1). If randomization fails for any of these variables, it will be added as a covariate to subsequent analyses. We will assess whether there are main effects of clinic site or interactions with study arm (ie, whether data can be pooled across sites). If data cannot be pooled across sites, the clinic site will be addressed either by multilevel modeling or by treating the clinic site as a moderator, depending on the analyses being run.

#### Outcome Assumptions

Normality, linearity, and homoscedasticity or homogeneity of variance for outcome data will be assessed using descriptive statistics and graphical representations. Data transformation, linear mixed models (LMMs), or nonparametric tests will be used to deal with the assumption failures of outcome data.

#### Missing Data

In previous work with older adults using ET, we kept missing data on core interview items to about 2%; we expect similar rates in this study. In primary care, data are not likely to be missing at random (ie, the probability that data are missing relates to what the data would have been had the data been observed). We will conduct a sensitivity analysis on missing data using logistic regression to examine whether dropout at follow-up is associated with observed or assigned factors, covariates, or outcomes at baseline [74]. If missing data affect power or are significantly not missing at random, LMMs or multiple imputation will be used [75].

#### Effectiveness of UC+ET Versus UC+Internet

Given that we expect our primary outcomes to be highly correlated, we plan to assess the effectiveness of UC+ET versus UC+internet on improving quality of life, psychological well-being, and loneliness using a repeated measures multivariate analysis of covariance (MANCOVA). If the continuous secondary outcomes (health or laboratory scores, symptom distress, and medication adherence) are highly correlated, they will be assessed using repeated measures MANCOVA. If not, we will run 3 separate repeated measures analyses of covariance (ANCOVAs). Secondary outcomes with count data (falls, symptom distress, medication adherence, crisis health care use, and long-term care use) will be assessed using repeated measures generalized linear mixed modeling (GLMM) with Poisson regression. Continuous exploratory outcomes (diet and problem drinking) will be assessed using repeated measures ANCOVAs or repeated measures MANCOVAs, depending on the level of correlation between these outcomes. Count exploratory outcomes (cigarettes per day and pain medication issues) will be assessed using repeated measures GLMM with Poisson regression.

#### ET Use

Within the UC+ET arm, we will conduct exploratory analyses on amount and type of ET use to describe patterns of use and test the effect of ET use on primary outcomes using LMMs.

#### Moderation

Moderators (scheduled health care use, number of chronic conditions, and sex) will be tested separately to determine whether study arm effects on the 3 primary and 2 of the secondary outcomes (falls and symptom distress) differ because of any of our hypothesized moderators. The same methods described earlier will be used with the addition of a moderator. For continuous moderators, LMM and GLMM are used instead of general linear models.

#### Mediation

The effect of study arm on mediators will first be tested using a repeated measures MANCOVA for anxiety and depressive symptoms and repeated measures ANCOVAs for health coping strategies, health-related motivations, and relatedness. Structural equation modeling will then be used to test mediation on primary and secondary outcomes examining those mediators that were significantly (*P*<.05) affected by study arm. Similarly, the effect of mediation will be tested only on outcomes that were significantly (*P*<.05) affected by study arm. All models will be tested as follows: study arm predicting outcome at 12 months mediated by mediator at 6 months, using linear regression for continuous outcomes, Poisson regression for count outcomes, or Zero-Altered Poisson regression for zero-inflated count data.

#### Type 1 Error

In cases where multiple tests relate to a single theoretical question, the Holm-Bonferroni method will be used to counteract the problem of multiple comparisons. For example, Holm-Bonferroni *P* value adjustments will be made to the 5 separate tests of individual health scores (mm Hg, mg/dL, HbA_1c_, BMI, and pain) when examining if patients assigned to UC+ET have greater improvements in individual health scores than patients assigned to UC+internet.

### Qualitative Analysis

A coding scheme of key themes will be constructed based on the research questions (perceived benefits, barriers to use) and examination of the data. Once reliability is established with 2 independent coders (minimum Krippendorff α of .80 per category), manifest expressions of benefits and barriers will be coded. More subtle themes, particularly regarding meanings and contexts of use, will be tagged for deeper qualitative analysis using NVivo (QSR International).

### Trial Registration and Funding

This study has been funded by the National Heart Lung and Blood Institute, National Institutes of Health (NIH), United States Department of Health and Human Services (grant number 1R01HL134146-01A1); received ethical approval from the University of Wisconsin Health Sciences Institutional Review Board (reference number 2017-0849) on September 11, 2017; and is registered at ClinicalTrials.gov (NCT03387735).

## Results

Recruitment was completed with 346 participants (target=330). Data collection is under way and to be completed in June 2021. The results will be communicated through publications and presentations.

## Discussion

### Changing Health Care Delivery

ET is conceived as an integrated, multiservice eHealth innovation aimed at changing in the following ways how care is delivered to patients coping with MCCs.

#### Single Disease Versus Multiple Diseases

Although information and communication technologies have shown promise in managing chronic conditions, most address a single disease (eg, tracking blood glucose for patients with diabetes). In contrast, ET offers interventions targeting behaviors that impact nearly all chronic conditions, such as social support, tracking of general health behaviors (eg, sleep, medication management), and relaxation and physical exercise resources (Table 1).

#### Single Intervention Versus Multiple Interventions

Many health care apps rely on a single tool, such as social networks. Despite having similar objectives, however, individuals benefit differently from various training and support. ET offers patients a broad choice of web-based training and support options, including tracking of health status and medication adherence, peer support groups and private messaging, web-based activities to promote social connection with other ET users, daily journaling with positive psychology prompts, guided relaxation audio, exercise videos for seniors with health conditions, a dynamic collection of quality health information, social and web-based games for pleasure and distraction, and data sharing with clinicians.

#### Complex Versus Simple

Many computer-based systems make extensive use of text and are complicated to navigate, and systems on smartphones are often challenging for older adults because of vision problems or tremors. ET’s web-based design is based on extensive feedback from older adults and best-practice design principles for this population (eg, uncluttered screens, large type, good contrast) [76] to ease the user experience.

#### Clinic Based and Periodic Versus Just-in-Time and Anytime or Anywhere

Most tracking and support offered in traditional health care is built around periodic onsite contact with physicians. Unfortunately, this model is at odds with addressing problems and questions as soon as they occur, and it fosters a reluctance to contact a doctor when earlier help could make a difference. ET provides patients with anytime or anywhere access, frequent assessments, and customized, protected interventions for just-in-time support.

#### Productivity-Based Yet Expensive Versus Evidence-Based Yet Lower Cost

Productivity and cost pressures limit the clinician’s time with patients [77,78]. In contrast, ET is an inexpensive and consistent yet customizable system that provides many evidence-based components of chronic care management.

### Sustaining Use

A critical issue with any eHealth system is attrition. For example, studies have reported that approximately 25% of users abandon a health app after a single use [79], the average retention rate is just 29% after 90 days [80], and almost half of a diverse (age, race or ethnicity, and income) national sample of app users reported abandoning an app [81]. Activity trackers (eg, Fitbit) and other wearable sensors show similar drop-offs [82,83]. The engagement problem is even higher for older adults [81,84].

Our earlier RCT of ET found high and sustained engagement compared with reports for other health apps [79-83]. In months 1 to 6, 88.3% (174/197) of all participants used ET for a mean of 44.84 days. Nevertheless, in just 4 months, pages viewed dropped by 53%. Interviews with older adult participants found 2 main reasons: (1) the small but myriad hassles of access (going to and turning on the computer, remembering and entering a password, opening a service, etc) and (2) physical limitations such as arthritis and impaired vision.

Overcoming such barriers to sustained, in-depth use is a critical challenge. The ET system reported in this paper includes features designed to ease use (eg, large fonts and clear navigation) and promote engagement (eg, discussion prompts, new content daily, interactive games), and amount and type of ET use are among our exploratory outcomes. One of our latest projects, an Agency for Healthcare Research and Quality–funded study in its initial design phase, will involve converting ET to a voice-activated system, using technologies such as Google Hub Max to further improve accessibility and ease of use.

### Impact on Public Health

The ET intervention is designed to simultaneously address hypertension, hyperlipidemia, diabetes, obesity, and arthritis, as well as underlying behavioral components. ET may also be used for almost any conditions that co-occur as MCCs. If our hypotheses about the benefits of ET are supported, this could point to a shift from care that is place based, focused on medical management, and periodic to nearly continuous care that is focused on helping patients manage their own conditions via a system built on proven principles of easy, effective behavioral interventions. The benefits to both quality of life and cost of health care are potentially broad and lasting.

## Appendix

Multimedia Appendix 1 Peer Review from Funding Agency.

## Abbreviations

ANCOVA: analysis of covariance
CHESS: Comprehensive Health Enhancement Support System
CRDS: Clinical Research Data Service
EHR: electronic health record
ET: ElderTree
GLMM: generalized linear mixed model
LMM: linear mixed model
MANCOVA: multivariate analysis of covariance
MCCs: multiple chronic conditions
NIH: National Institutes of Health
PROMIS: Patient-Reported Outcomes Measurement Information System
RCT: randomized controlled trial
REDCap: Research Electronic Data Capture
UC: usual care
UC+ET: usual care+ET access (experimental condition)
UC+internet: usual care+internet access but no ET (control condition)
UW: University of Wisconsin–Madison
