# Supplementary material for: A Web-Based eHealth Intervention to Improve the Quality of Life of Older Adults With Multiple Chronic Conditions: Protocol for a Randomized Controlled Trial
Source: JMIR Res Protoc. 2021 Feb 19;10(2):e25175. doi: 10.2196/25175 (PMC7935655; doi:10.2196/25175)
Supplement: Multimedia Appendix 1 [file resprot_v10i2e25175_app1.pdf]

SUMMARY STATEMENT

Release Date: **11/08/2016 Principal Investigators (Listed Alphabetically):**

GUSTAFSON, DAVID H. (Contact) MAHONEY, JANE E

Revised Date:

Applicant Organization: UNIVERSITY OF WISCONSIN-MADISON

Review Group:

Meeting Date: Council: Requested Start:

Project Title:

SRG Action: Next Steps: **Human Subjects: Animal Subjects: Gender: Minority:**

Children: Project Year

1

2
3
4
5 _______ TOTAL

BMIO
Behavioral Medicine, Interventions and Outcomes Study Section

10/17/2016 ***RFA/PA:*** **PA14-114 JAN 2017** ***PCC:*** **HHCP N 04/01/2017**

Heart-Related Multiple Chronic Conditions in Primary Care: Behavioral Technology

Visit http://grants.nih.gov/grants/next_steps.htm 30-Human subjects involved - Certified, no SRG concerns 10-No live vertebrate animals involved for competing appl. 1A-Both genders, scientifically acceptable
1A-Minorities and non-minorities, scientifically acceptable 3A-No children included, scientifically acceptable
Clinical Research - not NIH-defined Phase III Trial

Application Number:

1 R01 HL134146-01A1

2 BMIO

1 R01 HL134146-01A1

GUSTAFSON, D

1R01HL134146-01A1 GUSTAFSON, DAVID

RESUME AND SUMMARY OF DISCUSSION: This application proposes to test the C-CHESS computer technology for enhancing self-management in multiple chronic conditions by interacting with patients and updating their clinicians using supportive tools. The investigative team, environment, and innovation are excellent. They were very responsive to prior critiques and now the scientific premise, transparency, and reproducibility are strengthened. Other strengths include its duration and combination of behavioral and social elements, although the team C-CHESS content has not been developed yet. In summary, the committee concluded that this work could exert a sustained high overall impact on chronic care management and preventive cardiology.

DESCRIPTION (provided by applicant): This R01 proposal seeks to test a computer technology (C- CHESS) that offers a common approach to multiple chronic conditions (MCCs). Among Medicare beneficiaries, 65% have 3 or more MCCs and 23% have 5 or more. People with MCCs account for 90% of Medicare spending. MCCs are usually addressed in primary care. Increasing workloads in primary care often prevent clinicians from addressing self-management skills with patients, though such skills as goal-setting and tracking are important for managing chronic conditions. Behavioral interventions such as cognitive behavioral therapy, self-monitoring, and social support can improve both self- management skills and health outcomes, and technology can effectively deliver such interventions. C- CHESS (Chronic Condition Health Enhancement Support System) provides tools, motivation, and social support to help patients with MCCs better manage their conditions, including interactions that arise among the combination of conditions. C-CHESS is built on elements of success behavioral interventions: long duration, ongoing outreach, monitoring, prompts, action planning, problem solving, self-tailoring, and peer support. C-CHESS addresses clinician burden by promptly communicating important changes in patient status and—by improving patient-self-management—reducing primary care visits. C-CHESS will be tested in a randomized clinical trial in 5 primary care clinics in southern Wisconsin. The trial will involve 330 patients age 65 and older with 3 or all 4 of these conditions: hypertension, hyperlipidemia, diabetes, and osteoarthritis. Patients will be assigned to treatment as usual + C-CHESS or treatment as usual + access to the Internet. The trial will last 12 months with a 6- month follow up. The goal of the trial is to detect differences between the 2 groups in quality of life and use of primary care.

Secondarily, the trial aims to detect differences between the 2 groups in (1) measures for each condition (blood pressure, LDL, blood sugar, and pain); (2) a composite score that combines the individual measures; and (3) number of symptoms patients have from a list of 8. The trial will also examine factors that may mediate the relationship between the interventions and outcomes: adherence to medications and appointment attendance, as well as the 3 components of self- determination theory (patient competence, relatedness to others, and motivation). Finally, the trial will examine factors that may affect the strength of the relationship between C-CHESS and outcomes: gender, age, and number of chronic conditions. If successful, C-CHESS will improve the health and reduce the burden on primary care of a large, growing, expensive group of patients whose conditions are not now well addressed. Successful implementation may point to a shift from care that is place- based, focused on medical management, and periodic to care focused on helping patients manage their own conditions through a system built on the proven principles of effective behavioral interventions made easy for both patients and clinicians to use.

PUBLIC HEALTH RELEVANCE: Most adults 65 and older have multiple chronic conditions that relate to behavioral issues such as not taking medicines as prescribed. This project will test a computer- based system for older adults who have any three or all four of these conditions: hypertension, high cholesterol, diabetes, and arthritis. The system is designed to improve patients' quality of life and reduce their use of primary care by helping them better manage their own conditions through information, support, and motivational aids provided in the computer system called C-CHESS.

3 BMIO

1 R01 HL134146-01A1

GUSTAFSON, D

CRITIQUE 1:

Significance: 2 Investigator(s): 2 Innovation: 2 Approach: 3 Environment: 1

Overall Impact: This is a revised application from a well-established investigator to test the C-CHESS system, a mhealth application designed to assist geriatric patients with multiple chronic conditions (mcc), specifically hypertension, hyperlipidemia, diabetes and OA. The application is significant in that mcc is prevalent among older adults, and will continue to grow in prevalence as the population continues to age. In addition to its high relevance to primary care, the mhealth platform makes it highly scalable if successful. The investigative team is top notch and highly experienced, led by a PI who is a member of the National Academies of Science. The team has performed several similar studies using the CHESS system for various conditions with great success. C-CHESS will build upon an application (Elder Tree) that is currently being tested. The innovation level is high, in multiple ways, including the mhealth platform that focuses on multiple conditions, the tailoring to older populations, behavioral and informational tools for the patients and a patient centered report for clinicians. The approach is sound, utilizing a randomized design in 5 clinics in an academic health system. The revised application addresses this reviewer’s concern about lack of preliminary evidence. The investigators now include a preliminary evidence of efficacy of the CHESS platform in elderly patients with the chronic conditions of interest. The environment is excellent. Overall, there is enthusiasm for this strong

revised application, which appears to have the potential to exert a significant impact on the field of geriatric medicine in the future.

1. Significance: Strengths

- • This is a revised application from a well-established investigator to test the C-CHESS system, a mhealth application designed to assist geriatric patients with multiple chronic conditions, specifically hypertension, hyperlipidemia, diabetes and OA. These are important risk factors for the leading cause of death in the US.
- • The application is significant in that mcc is prevalent among older adults, and will continue to grow in prevalence as the population continues to age. In addition to its high relevance to primary care, the mhealth platform makes it highly scalable if successful.
- • The application is significant in that mcc is prevalent among older adults, and will continue to grow in prevalence as the population continues to age. In addition to its high relevance to primary care, the mhealth platform makes it highly scalable if successful.

Weaknesses

• None noted

2. Investigator(s): Strengths

- • The investigative team is top notch and highly experienced, led by a PI who is a member of the National Academies of Science.
- • The team has performed several similar studies using the CHESS system for various conditions with great success.

• Roles well defined and justified

Weaknesses

• None noted

3. Innovation: Strengths

• The innovation level is high, in multiple ways, including the mhealth platform that focuses on multiple conditions, the tailoring to older populations, behavioral and informational tools for the patients and a patient centered report for clinicians.

Weaknesses

• None noted

4. Approach: Strengths

- • Randomized design in 5 clinics in an academic health system Is a strength
- • Period of pilot testing and refinement based on patient and clinician feedback is a strength
- • Detailed Analytic plan
- • Long follow up period, including a plan to examine long term effects six months after intervention is withdrawn

Weaknesses

- • While the proposed project is patient and community oriented, and may have some CBPR- informed components, the proposal is not CBPR. True CBPR requires that the target community give input from the outset.
- • How will 'action' thresholds for the clinician reports be tailored/individualized?
- • Intervention will be developed from existing Elder Tree, which is currently being tested. Seems

prudent to build onto the elder tree platform AFTER we know that it works.

5. Environment: Strengths

• The scientific and clinical environments are excellent

Weaknesses

• None noted

Protections for Human Subjects:

Acceptable Risks and/or Adequate Protections
Data and Safety Monitoring Plan (Applicable for Clinical Trials Only):

Acceptable

Center for Health Enhancement Systems Studies has an independent Data Monitoring Committee

5 BMIO

1 R01 HL134146-01A1

GUSTAFSON, D

Inclusion of Women, Minorities and Children:

- • Sex/Gender: Distribution justified scientifically

- • Race/Ethnicity: Distribution justified scientifically
- • Inclusion/Exclusion of Children under 18: Excluding ages < 18 justified scientifically
- • focus on geriatric population with mcc

Vertebrate Animals:

Not Applicable (No Vertebrate Animals)

Biohazards:

Not Applicable (No Biohazards)

Revision:

• Highly responsive to critiques **Budget and Period of Support:**

Recommend as Requested

CRITIQUE 2:

Significance: 3 Investigator(s): 2 Innovation: 2 Approach: 3 Environment: 3

Overall Impact: This revised proposal work is likely of very good overall impact. Its objective is to test the C-CHESS computer technology for enhancing self-management in multiple chronic conditions by interacting with patients and updating their clinicians using an array of supportive tools. Indeed, an automated system that simultaneously handles these comorbidities could have a significant impact upon primary care. In this revision scientific premise, transparency, and reproducibility are now strengthened by the inclusion of new preliminary data on the Elder Tree ET intervention showing reasonable effect sizes on primary outcomes. The envisioned C-CHESS intervention may also facilitate the transition to population health. Other strengths include its duration and combination of behavioral and social elements. However the team C-CHESS content has not been developed yet, and it only shares the platform with the pilot intervention. However, the team argued that the intervention was a tool, and that its content was secondary. Methodological rigor is evident from several angles, with a fuller description of the envisioned C-CHESS intervention, careful patient-centered development to reduce technology overwhelm, reproducibility potential and methodological transparency, and plans to address relevant biological variables. Effect sizes were presented for a small subset of the ELDER-TREE study, but this prior work seems to target psychosocial isolation rather than health behaviors. Despite these limitations, the strength and experience of the team increase the chance that this work could exert a sustained, powerful influence on the chronic care management and preventive cardiology.

1. Significance: Strengths:

- • The objective is to test a computerized approach (C-CHESS; chronic condition health enhancement support system) for enhancing the self-management of the combination at least 3 of these 4 conditions: hypertension, hyperlipidemia, diabetes, and osteoarthritis. An automated system that can simultaneously handle common comorbidities could have a significant impact upon primary care, where clinicians seldom have the time or training to focus upon self- management.
- • Scientific premise, transparency, and reproducibility are now significantly strengthened by the inclusion of new preliminary data on a similar intervention (Elder Tree ET, n=65) in same population, indicating reasonable effect sizes on primary outcomes.
- • Inclusion of selected CBT strategies, sustained duration, prompting, self-tailoring, and anonymized peer support.
- • C-CHESS seems promising, comprehensive, and potentially cost effective because it will provide patients with structure and support while maintaining appropriate clinician involvement. It may even facilitate the transition to population health.
- • The management of multiple chronic conditions is of importance to Medicare and the US public health in general. Specifically, hypertension, hyperlipidemia and obesity, which are collectively responsible for a large proportion of morbidity and mortality.
- • The user-centered and simple interface is based upon information form focus groups, home visits and standardized assessments, and thus likely to be well accepted.
- • The C-CHESS intervention is long-duration and includes outreach, monitoring, prompts, action planning, problem solving, self-tailoring, and peer support.

Weaknesses:

• C-CHESS intervention is not yet developed.

2. Investigator(s): Strengths:

- • The Contact PI, Dr. Gustafson is a professor of Industrial Engineering at Univ. Wisconsin, directs the Center for Health Enhancement Systems Studies and an AHRQ Aging Center, directed several prior CHESS projects, and has published widely on interactive behavioral health technology. In sum, he is very well-established investigator and ideally qualified to direct this project.
- • The co-PI Dr. Mahoney is a U.W. geriatrician and frequent collaborator of Dr. Gustafson's. Her primary responsibility will be clinical oversight of trial participants.
- • The investigators have complementary and integrated expertise. The roster includes a clinical liaison (Brown) and expertise in experimental design, psychometrics, and statistics (Shah).
- • A reasonable multiple PI plan that specifies project-appropriate organizational structure with delineated roles, and processed for scientific decision-making, communication, and conflict resolution.

Weaknesses:

• None noted.

3. Innovation: Strengths:

- • The intervention fills an important scientific and clinical gap, because it simultaneously addresses up to 4 common chronic conditions. This could significantly advance the interface of health IT and primary care management.
- • Instrumentation: Variety of interaction modalities increases usability by older patients, whereas many interventions have limited channels of communication such as text or web alone.
- • Additionally, it is a major advantage over existing systems to tailor the intensity parameters by patient preference and specific condition(s).
- • Novel social support element to address loneliness in the context of aging with chronic disease.

Weaknesses:

- • None noted.

4. Approach: Strengths:

- • Methodology and analyses are well-reasoned for meeting the clearly specified specific aims.
- • Scientific rigor enhanced by randomization of 330 patients with multiple conditions across 5 primary care clinics, and 12-month duration with by 6 month follow-up. Clear but realistic entry criteria. C-CHESS intervention is much better described than previously.
- • Proposal was strengthened by inclusion of stronger control arm including web access and training.
- • Assessments and data analytic plans are well-documented, bolstering research rigor.
- • Careful patient-centered development to ensure acceptability/usability and reduce the risk of technology overwhelm despite a multifaceted program. Considerable development of base intervention with patient input to make it simple and acceptable. Related, patient control over choice of tools, prompt frequency, pacing, etc.
- • Methodological transparency enhanced by consideration of problems, alternatives, and benchmarks plus adequate specification of procedures and data analysis.
- • Adequate plans to address relevant biological variables affecting outcomes, including gender, age, and condition-specific outcomes (e.g., HbA1c for patients with diabetes).

  Weaknesses:

  - • Although the envisioned C-CHESS intervention was far better described, the fact stands that it has not yet been pre-tested let alone even developed. In fact almost the entire first year was needed for its development. The team argues that that the intervention content is secondary.
  - • Effect sizes were presented for a small subset of the Elder Tree study, which seems to have targeted psychosocial isolation and not self-management. The

pilot data do not correspond ideally with the proposed intervention, which is yet to be developed.

5. Environment: Strengths:

• The University of Wisconsin – Madison, CHESS center, and 5 clinics seem very well equipped to host the proposed project. This should contribute to the probability of success. Adequate institutional support, equipment other resources are available.

Weaknesses:
• None noted.

Protections for Human Subjects:

Acceptable Risks and/or Adequate Protections

• Risks include confidentiality breach, psychological stress due to certain personal nature of items, and typical risks associated with internet use. Protections include informed consent, participant education, voluntary participation/continuation, and federal certification of confidentiality. These seem adequate. The research potentially has direct and indirect benefits, and seems ethically justified.

Data and Safety Monitoring Plan (Applicable for Clinical Trials Only): Not Applicable (No Clinical Trials)

Inclusion of Women, Minorities and Children:

- • Sex/Gender: Distribution justified scientifically
- • Race/Ethnicity: Distribution justified scientifically
- • For NIH-Defined Phase III trials, Plans for valid design and analysis: Not applicable
- • Inclusion/Exclusion of Children under 18: Excluding ages <18; justified scientifically
- • 55% of participants are projected to be female. No participants will be under 18. Over-recruiting so that 13% will be African-American, and 20% Hispanic/Latino.

Vertebrate Animals:

Not Applicable (No Vertebrate Animals)

Biohazards:

Not Applicable (No Biohazards)

Budget and Period of Support:

Recommend as Requested

CRITIQUE 3:

Significance: 2 Investigator(s): 1 Innovation: 2 Approach: 4 Environment: 1

9 BMIO

1 R01 HL134146-01A1

GUSTAFSON, D

Overall Impact: This is a resubmission of a R01 proposal to test an e-health application, C-CHESS in 330 patients 65 years or older with multiple chronic conditions. The scientific premise of this proposal is sound as there is ample pilot data from the investigative team using a similar approach. The investigative team, environment, and innovation of the proposal are excellent. The focus on quality of life and health care utilization enhance the significance of the research as these are patient-centered and appropriate outcomes for the type of intervention and multiple chronic conditions in the proposed study. Changes to this revised application include modification of the test populations that are based on pilot data, so that is a substantial improvement in the research plan and adds to the scientific rigor of the proposal. The choice of comparison group, while improved since the previous application, is less rigorous as there is no evidence that simply providing access to the internet might be a useful intervention in these populations. While there is no way to equalize the number of contacts patients have across interventions, some type of required interaction with a particular website would have been preferable. Finally, the sampling of a predominantly Caucasian sample will limit the translation of this intervention system to practice.

Protections for Human Subjects:

Acceptable Risks and/or Adequate Protections

• The potential benefits of this minimal risk study outweigh the possible risks involved for participants.

Data and Safety Monitoring Plan (Applicable for Clinical Trials Only): Acceptable

o DSMPincludesamonitoringcommitteeandsafetyofficerthatarepartoftheCenterfor Health Enhancement Systems Studies

Inclusion of Women, Minorities and Children:

- • Sex/Gender: Distribution justified scientifically
- • Race/Ethnicity: Distribution justified scientifically
- • For NIH-Defined Phase III trials, Plans for valid design and analysis: Not applicable
- • Inclusion/Exclusion of Children under 18: Excluding ages <18; justified scientifically

- • Sampling of racially diverse participants is anticipated to be quite low, which is a substantial shortcoming of the proposal as it impacts translation to practice.

Vertebrate Animals:

Not Applicable (No Vertebrate Animals)

Biohazards:

Not Applicable (No Biohazards)

Resubmission:

• The investigative team was generally responsive to prior critiques.

Budget and Period of Support:

Recommend as Requested

THE FOLLOWING SECTIONS WERE PREPARED BY THE SCIENTIFIC REVIEW OFFICER TO SUMMARIZE THE OUTCOME OF DISCUSSIONS OF THE REVIEW COMMITTEE, OR REVIEWERS' WRITTEN CRITIQUES, ON THE FOLLOWING ISSUES:

PROTECTION OF HUMAN SUBJECTS (Resume): ACCEPTABLE
INCLUSION OF WOMEN PLAN (Resume): ACCEPTABLE
INCLUSION OF MINORITIES PLAN (Resume): ACCEPTABLE
INCLUSION OF CHILDREN PLAN (Resume): ACCEPTABLE
COMMITTEE BUDGET RECOMMENDATIONS: The budget was recommended as requested.

Footnotes for 1 R01 HL134146-01A1; PI Name: GUSTAFSON, DAVID H.

NIH has modified its policy regarding the receipt of resubmissions (amended applications). See Guide Notice NOT-OD-14-074 at http://grants.nih.gov/grants/guide/notice-files/NOT-OD- 14-074.html. The impact/priority score is calculated after discussion of an application by averaging the overall scores (1-9) given by all voting reviewers on the committee and multiplying by 10. The criterion scores are submitted prior to the meeting by the individual reviewers assigned to an application, and are not discussed specifically at the review meeting or calculated into the overall impact score. Some applications also receive a percentile ranking. For details on the review process, see http://grants.nih.gov/grants/peer_review_process.htm#scoring.

Response to Reviewer Concerns Regarding Grant Number: 1R01HL134146 - 01A1

David H. Gustafson PhD and Jane Mahoney MD
University of Wisconsin – Madison
608 239-5535
dhgustaf@wisc.edu

We are happy the reviewers praised: our investigative team, environment, innovation, responsiveness to prior critiques, scientific premise, transparency, reproducibility, the study plan's duration, combination of behavioral and social elements, the promise of CHESS platform and that "this work could exert a sustained high overall impact on chronic care management and preventive cardiology".

We also appreciate the concerns raised by the reviewers and respond to them below.

- *True CBPR requires that the target community give input from the outset.* We apologize if we incorrectly applied the CBPR label. We engaged hundreds of older adults in designing Elder Tree. Since we received the reviewer comments we have gone farther. Four new groups of paid older adults suggested ways to refine this proposal. *Culture Advisors* - 15 minorities (6 Native Americans) suggested ways to adapt C-CHESS to their cultures and get older adults to use C-CHESS. *Patient Advisors* - 4 patients with MeS (all minorities) described their challenges with MeS. *Study Advisors* - 9 ET users (5 minority) suggested ways to describe the project and recruit older adults, how to create community, how to compensate the comparison group, and what outcomes are most crucial. From these groups, we identified 7 older adults to serve on a new steering committee and continue to advise us on study implementation. Changes we propose, based on our advisors' input include:
  - *More community:* A key focus was on creating community to combat isolation and support self-management. Following our advisor's advice, we will add games that participants can play together (e.g., Words With Friends) and that we encourage buddy systems where individuals could share their self-management goals and successes (e.g., steps walked).
  - *More happiness:* Advisors wanted C-CHESS to bring them happiness and fun, not just reminders about obligations. We will add a joke of the day, a photo of the day (e.g., scenes of nature) have greater emphasis on shared games (see above) and on meditation.
  - *Fairness:* Our older adult advisors (and reviewers) wanted us to compare C-CHESS to a viable alternative (not just to treatment as usual), so patients will now have access to computers and websites in both arms of the study.
  - *Building trust:* We will engage widely known and trusted local and national figures to endorse the project to support study recruitment.

  Other advice had already been included in C-CHESS design.
  - *Privacy***:** Older adults will have the option of *not* sharing health-tracking data with their clinical team.
  - *Help finding local resources:* Sections of C-CHESS provide links to resource centers such as the local Aging and Disability Resource Centers near our study.
  - *C-CHESS is a walled garden*. Participants must be ≥65 and vetted. The site has no ads and nothing is sold.
  - *Ease of use:* The computer has a touchscreen, not a mouse. Videos show how to use each service.
  - *Warmth***:** When new people join, they will get a personal welcome. Everyone who posts a message gets a response.
- *Testing of elder Tree is not yet complete.* The randomized trial testing of Elder Tree is now complete with 396 adults ≥65. Of these, 103 had metabolic syndrome (MeS): 53 had ET, 50 were controls. The primary endpoint for analysis was impact at 12 months.
  - *Use of ET* was high and sustained, even though almost 50% of participants did not previously have a computer or internet access. Of 203 participants in the ET arm, only 2 did not use the system at all. Almost 75% of participants in the ET arm posted content; posting 5803 messages (3272 discussion groups, 2531 private messages). Participants turned on ET an average of 15.3

times per month. ET was successful at engaging participants with relatively little education: Of the 20% of participants who posted most often in the discussion group, only 18% had a college degree and only 26% had their own computer before the study. ET was used most by people who were vulnerable and isolated. Comparing the 20% "most frequent posters" to the other ET participants, 88% of frequent posters (vs. 54%) lived alone, 67% (vs. 26%) had fallen recently, 60% (vs. 40%) felt depressed, 34% (vs. 10%) had trouble walking at home, 8% (vs. 31%) had someone to turn to for personal problems, and only 26% (vs. 44%) had good quality of life.

- *Value of ET*: We used data from pretest and 12 months to evaluate ET effects on quality of life, mood, support, symptom distress, and use of non-primary care health services (e.g., hospital admissions). MANOVA indicated a statistically significant interaction between MeS and ET on the dependent variables, p=.008. Univariate results revealed significant interactions for 3 of 6 outcomes, such that among the 103 with MeS (53 with ET vs. 50 controls), ET had significant effects on quality of life, p=.012; symptom reduction, p=0.003; and health service use reduction, p=.015. Effect sizes (ES) were: QoL, .35; symptom distress, .68; use of primary care, .36; and all health service use, .39.
- *Additional qualitative evidence* of value comes from participants' posts. One wrote, "*I've learned a lot from the program. You can talk and state your opinions and someone will help you. There are so many friendly people on here. I still don't know much about the computer but I get by. It keeps me from being lonely, gets me to keep in touch, and keeps me busy. You get to learn more every day.*" (posted 3/16/15 at 7:14 AM). Another wrote, "*I am so touched by your post...and everyone's post. We truly are a family. We help each other, offer advice whether asked for or not, disagree, care about each other, laugh, hug & do everything a family does. It is a wonderful family to belong to!! I am so grateful*." (posted 10/23/14 at 11:05 AM).

- *Effect sizes were presented for a small subset of the Elder Tree study, which seems to have targeted psychosocial isolation and not self-management.* Behavioral change was not a goal of this study; quality of life was. Hence we did not collect behavior change data. We have studied behavior change in other research such as smoking and asthma (where we found a positive dose response impact of adding CHESS to a medication intervention) but subjects were younger. We are happy to include behavior changes (smoking cessation, physical activity, weight, stress, healthy eating) as exploratory outcomes. Below are measures that we could add to our surveys (comments below are taken from Glasgow et al.[1])

- *Healthy Eating.* The Summary of Diabetes Self-Care Activities (SDSCA)[2] includes 4 dietary items low fat, high fiber, and increased fruit and vegetable intake. SDSCA has been widely used and found to be sensitive to change in intervention studies with diabetes patients. Questions seem applicable to non-diabetic adults.
- *Physical Activity.* The Rapid Assessment Physical Activity Scale (RAPA)[3] has 9 yes/no items assessing type and amount of physical activity. Clinicians and measurement experts at the University of Washington developed this scale for primary care settings. One advantage is that it enables respondents to visualize differences in activity intensity.
- *Risky Drinking.* Three items from the BRFSS.[4] These items offer a fast, simple way to detect risky drinking and to examine changes in binge drinking. Findings can be compared with national norms and related to Healthy People 2010 goals.
- *Smoking.* We would use three items from national health surveys to assess whether respondents ever smoked, their current smoking status, and extent of smoking. The recommended items allow tracking on Healthy People 2010 goals.

- *The comparison group: there is no evidence that simply providing access to the Internet might be a useful intervention in these populations. Some type of required interaction with a particular website would be preferable.* The intent of the comparison group was to offer an attention control and not to conduct a comparative effectiveness study. There are websites that could be linked. They offer good information about MeS such as ones developed by:
  - NHLBI (https://www.nhlbi.nih.gov/health/health-topics/topics/ms),

- The American Heart Association (https://www.heart.org/HEARTORG/Conditions/More/MetabolicSyndrome/Metabolic-Syndrome_UCM_002080_SubHomePage.jsp) and
- The Mayo Clinic http://www.mayoclinic.org/diseases-conditions/metabolic-syndrome/home/ovc-20197517

These websites provide interesting comparisons because C-CHESS is designed around providing tools to improve social relatedness, motivation and coping competence. C-CHESS intentionally places less stress on disease specific content, consistent with the FOA's call for a "common conceptual model" (regardless of the chronic conditions chosen). For us, providing information is secondary, in part because other research suggests that information alone is unlikely to create behavior change[5] and because a lot of good information (see above) is already available and "linkable" through C-CHESS". We are quite willing the change the comparison group (and C-CHESS for that matter) to link these websites to our computers' desktops for easy access. We hope that this paragraph addresses a related matter, where reviewers expressed concern that *C-CHESS content has not been developed yet.*

- *The sampling of a predominantly Caucasian sample will limit the translation of this intervention to practice*. It is true that Madison's population is primarily white non hispanic (about 90%). One of our sites is a Federally Qualified Health Center, which serves low-income people, including a larger proportion of minorities. We will oversample minorities to add as many as possible to our study with the goal of achieving 20%, and divide them equally between experimental control groups. This would yield about 30 minorities in each arm of the study, enough to obtain a preliminary sense of differential impact in this subgroup.
- *Almost the entire first year is needed for C-CHESS development*. We apologize for our lack of clarity. We will begin recruiting in month 9. During the first 8 months, a number of activities will take place including establishing the study team, finalizing protocols, introducing the study to clinic staff, refining C-CHESS itself but most of all obtaining, final IRB approval. We have already started that process, but cannot guarantee that it can be completed much before that time. However, we will use that time well, to ensure an efficient implementation.

---

[1] Glasgow R, Ory M, Klesges L, et al. (2005). Practical and relevant measures of patient health behaviors for primary care research. *Ann Fam Med.* 3(1): 73-81.

[2] Toobert DJ, Hampson SE, Glasgow RE. The summary of Diabetes Self-Care Activities Measure: results from seven studies and revised scale. Diabetes Care. 2000;23:943–950.

[3] http://hmcrc.srph.tau.edu.

[4] U. S. Department of Health and Human Services. Behavioral Risk Factor Surveillance System. 2004.

Available at: http://www.cdc.gov/brfss/index.htm

[5] http://www.health.harvard.edu/staying-healthy/why-its-hard-to-change-unhealthy-behavior
